# Humoral Immune Response in Calves Vaccinated with Monovalent Vaccines or a Trivalent Combination Thereof and Matching of These Vaccines to the Selected Circulating Foot-and-Mouth Disease Viruses in Ethiopia

**DOI:** 10.3390/vaccines11081352

**Published:** 2023-08-10

**Authors:** Fanos Tadesse Woldemariyam, Demessa Negessu, Tsion Bilata, Ayelech Muluneh, Dereje Shegu Gebreweld, Ibsa Teshome Ebisa, Jan Paeshuyse

**Affiliations:** 1 Laboratory of Host-Pathogen Interaction in Livestock, Division of Animal and Human Health Engineering, Department of Biosystems, KU Leuven, 3000 Leuven, Belgium; fanos.tadesse@aau.edu.et; 2 College of Veterinary Medicine, Addis Ababa University, Bishoftu P.O. Box 34, Ethiopia; 3 Animal Health Institute, Sheger City P.O. Box 04, Ethiopia; 4 Department of Veterinary Laboratory Technology, Guder Mamo Mezemir Campus, Ambo University, Ambo P.O. Box 19, Ethiopia

**Keywords:** ELISA, antibody, cattle foot-and-mouth disease, vaccine matching, vaccination, neutralization test

## Abstract

Foot-and-mouth disease (FMD) is an endemic, highly contagious, and devastating disease of livestock production in Ethiopia. Control of this disease relies mainly on prophylactic vaccination by willing farmers without a countrywide vaccination program. The objectives of this study were to quantify the humoral immune response and evaluation of the serological relationship of the vaccine strain used with representative field strain isolates. This was performed by primo vaccination of 6–9-month-old Holstein Friesian calves (35 treatment and 4 control calves) on day one and booster vaccination on day 28. Calves were vaccinated using the locally available National Veterinary Institute (NVI), Bishoftu, Ethiopia, inactivated aluminum hydroxide adjuvant monovalent (either O, A, SAT-2 alone) or trivalent (combination of A, O, SAT-2) vaccine (A/ETH/6/2000 (G-VII, O/ETH/38/2005(EA-3) and SAT-2/ETH/64/2009(XIII)). A 2 mL or 4 mL dose was used to vaccinate all calves except the animals that served as a control. In the case of the trivalent vaccine, a 4 mL dose was used to vaccinate calves. The serum was collected at 7, 14, 21, 28, and 56 days post-vaccination (d.p.v.). The humoral immune response was quantified by the solid-phase competitive enzyme-linked immunosorbent assay (SPC ELISA) and the virus-neutralization test (VNT). The serological relationship of heterologous and homologous viruses was also evaluated by adjuvant vaccine matching tests. The r1-value was determined using serum collected 21 d.p.v. An increase in immune response was observed from 7 d.p.v. to 28 d.p.v. in calves who received a 4 mL dose containing a 10^7.24^ antigen load of 100 tissue culture infective dose (100TCID50) virus titer in the formulation. Upon receiving a booster dose on day 28, the humoral immune response was checked on the 56th day post-initial vaccination. Amounts of 54%, 72%, 79%, and 72% of inhibition for A, O, SAT-2, and trivalent vaccine in the three serotypes SPCE, respectively, was measured. Here, it was found that the immune response of calves increased from day 7 to 56, as evidenced by SPCE analysis. Likewise, an increase in antibody titer measured by a one-dimensional virus neutralization assay was also in line with SPCE analysis. This indicates that the vaccine is capable of inducing a neutralizing antibody that confers a protective immune response in 70%, 62%, and 100% heterologous field strains of A, O, and SAT-2 isolates, respectively, and has an average antigenic relationship of >0.3 with a standard deviation of +0.05 (N = 3) to the vaccine strains A/ETH/6/2000, O/ETH/38/2005 and SAT-2/ETH/64/2009, respectively, when using the one-dimensional virus neutralization test. The contribution and importance of this study is a confirmation of the vaccine and the field strain serological relationship for serotype SAT-2 strain and further research/change of vaccination strategy/ improvement in the currently used vaccine to cover a wide range of prevailing genotypes/lineages and induction of sound immune response after vaccination for serotype A and O strain. This study suggests that the trivalent vaccine produced by the National Veterinary Institute containing viral isolates from serotype O, A, and SAT-2 has a good serological relationship with the majority of circulating field strains in Ethiopia.

## 1. Introduction 

Foot-and-mouth disease (FMD) is caused by a positive-sense RNA virus called FMD virus (FMDV), of the genus *Aphthovirus* and the family of *Picornaviridae* [1]. FMD is endemic, contagious, devastating and affects a wide range of domestic and wild cloven-hoofed animals in many parts of the world [2,3]. Globally, seven FMDV serotypes (A, O, C, Southern African Territories (SAT)-1, -2, -3, and Asia1) were known to circulate [4] but serotype C circulation has never been reported since 2004 and is believed to be extinct [5]. Currently, serotypes A, O, SAT-2, and SAT-1 are endemic in Ethiopia [6,7,8,9]. This disease has direct (i.e., reduced milk yield, loss of draught power, retardation of growth, abortion in pregnant animals, and death in calves and lambs) and indirect economic impacts (i.e., restrictions on the trade of animals and animal products) mainly in endemic countries [10].

Vaccination is the most frequently used method to control FMD in an endemic situation, mainly in resource-poor countries which are unable to compensate for the costs related to culling [11]. However, control of FMD by vaccination is difficult due to genetic variation between the serotypes of FMDV and even within a serotype, the presence of antigenic subtypes can make it difficult to achieve protection by serotype-specific vaccination. In addition, vaccination needs to be coupled with the movement restriction of animals, as reviewed in [2]. Natural infection and vaccination induce a rapid humeral immune response, slightly faster in a vaccination compared to natural infection [12]. The short duration of immunity (about 6 months), lack of providing sterile immunity or failure to block the development of carriers, instability and the need of cold chain are some of the problems encountered with the current FMDV vaccines [13]. Further, being inactivated, non-replicative virus particles, low antigen payload/low concentration of immunogen, and the elimination of non-structural proteins (NSPs) during the antigen purification process are also problems associated with FMDV vaccines [14]. Most FMD vaccines contain inactivated purified antigens (inactivated virus), usually by chromatographic purification, and formulated with an adjuvant that differs between formulations [15]. Further, immuno-stimulants, the likes of C-type lectin and Toll-like receptor (TLR) as composite adjuvants, were shown to enhance the immune response [16], and the use of oil adjuvant together with aluminum hydroxide gel for vaccine preparation was shown to increase the efficacy of the vaccines [17].

In spite that the National Veterinary Institute (NVI) in Ethiopia changed the bivalent vaccine (O/281 and A/110) formulation into a trivalent (A/ETH/6/2000, O/ETH/38/2005, SAT-2/ETH/64/2009) formulation in 2009, FMD cases still occur in different parts of Ethiopia [18,19].

The NVI vaccine was formulated as a formalin-inactivated (0.3%), aluminum hydroxide gel [Al(OH)_3_] concentrated with saponin as an adjuvant (https://www.nvi.com.et/products/vaccines-against/ruminant-and-equine-diseases/fmd-2/, accessed on 25 June 2023) in spite of many researchers recommending oil-based adjuvants due to their long-lasting immunity and their stimulating of both the cellular and humoral immune response [17].

This raised concerns about the antigenic match and effectiveness of the FMDV vaccine strains used and if they are comparable to the circulating FMDV viral strains. Additionally, lack of well-organized nationwide vaccination strategies (quality, coverage, and timing) and the presence of the free animal movement without certification are the main factors that increase the occurrence and spread of FMD throughout the cattle market chain. To the best of our knowledge, limited studies have been conducted to evaluate the serological relationship between the vaccine strains and the field strains in Ethiopia. Two studies of Tesfaye et al. [20,21] documented the serological relationship between the field strain with the currently used vaccine strain using a one-dimensional virus-neutralization test (1dm-VNT) for serotype O and A that covered heterologous viruses of central Ethiopia and spanned an outbreak period of two years. To apply an organized control strategy, the capacity of the locally produced vaccines to induce an immune response, as well as the serological relationship between recently circulating viruses and the vaccine strain in use, should be identified. The vaccine immune response should be quantified in terms of serotypes, days, batches, and dose. This is mainly necessary to avoid a poor antigenic match between the circulating strain and the vaccine strain. This study tried to check the humoral immune response of calves vaccinated and the neutralizing capacity of antibody to the heterologous circulating field strains of FMDV. Therefore, this study aimed at the following:

Measuring the quantity of antibody induced against FMD in naïve calves vaccinated using either a monovalent or trivalent vaccines (A/ETH/6/2000 (G-VII), O/ETH/38/2005 (EA-3) and SAT-2/ETH/64/2009 (XIII)).

In vitro evaluation of the antigenic serological relationship of vaccine strain and selected representative field isolates of FMDV serotypes (A, O, SAT-2) using a one-dimensional virus-neutralization test (1dm-VNT).

## 2. Material and Methods

### 2.1. Ethics Statement

The Addis Ababa University College of veterinary medicine and agriculture Ethical Review Committee’s management standards were followed for all animal research (Ref. number VM/ERC/03/12/2016). All of the animals utilized in this investigation were safely bled.

### 2.2. Study Design and Sampling

Briefly, thirty-nine (35 treatment and 4 control) Holstein Friesian calves between 6 to 9 months of age (100–150 kg) were recruited and grouped. Then, these animals were screened for the presence of antibodies against non-structural proteins (NSP), indicative of previous infection with FMDV, using the PrioCHECK^®^ FMDV NSP according to the manufacturer’s instructions (https://www.thermofisher.com/order/catalog/product/7610440, accessed on 18 April 2023). The NVI vaccine from Bishoftu, Ethiopia, was used for this experiment. This vaccine was formulated as a formalin-inactivated, aluminum hydroxide gel [Al(OH)_3_] concentrate with saponin adjuvant. The monovalent or trivalent vaccine was formulated at an antigen/immunogen concentration of 10^7.24^ per milliliter of the dose. There were seven treatment group (five calves per group and one control group (four calves) in the experimental set up. Calves were vaccinated using monovalent (containing serotype A, O, SAT-2 in a separate preparation and trivalent vaccines (containing the three serotypes together) (see Table 1). These vaccines were administered subcutaneously using a 1.5 inch needle of 18 gauge, preferably in the dewlap region at day zero and day 28 (booster vaccination). Seven, fourteen, twenty-one, twenty-eight and fifty-six days following the initial vaccination, 7 mL of blood was collected from the jugular vein (Table 1). These samples were allowed to clot on the bench overnight at an inclined position and the serum was decanted on the next day [22]. Approximately 2 mL of serum was stored in a cryovial at −20 °C until further analysis.

### 2.3. Antiserum Preparation

Briefly, calves were vaccinated with a full dose (4 mL) or half (2 mL) dose of monovalent and or trivalent vaccines produced at the National Veterinary Institute, Bishoftu, Ethiopia. Post-vaccinal calf antiserum of days 14, 21, 28 and 56 d.p.v. was obtained and pooled. This was used for the virus neutralization test as recommended by WOAH [23]. Specifically, for the vaccine matching test, post-vaccinal serum of day 21 was used. Collected post-vaccinal serum was filtered with 0.22 µm filter and the complement protein was inactivated at 56 °C in a water bath for 30 min. The serum samples were titrated in two-fold dilution (test and control sera) 1/2 with media without serum using Eppendorf tubes. The final dilution to be transferred to the plate was prepared by diluting this to ¼ with media without serum.

### 2.4. Titration of Viruses

Dulbecco’s modified Eagle medium was used for the cultivation of Baby Hamster Kidney-21 (BHK)-21 adherent cells (kind gift from AHI, Sebeta, Ethiopia). The cells were cultured at 37 °C in an incubator with 5% CO_2_ supplied with 10% fetal bovine serum (pH 7.4).

For the vaccine-matching test, circulating field strains, preferably more than one representative isolate of serotype A, O, and SAT-2, were selected. The vaccine strains were chosen because they are the only vaccines available in Ethiopia. A one-dimensional virus-neutralization test was used to perform the vaccine-matching test according to previous recommendations [24,25,26]. Three independent virus-neutralization tests on different days were conducted to compute the average r1-value for each test virus as recommended by [27,28]. The three vaccine strains and representative field virus isolates were tittered by serial tenfold dilution using a tissue culture microtiter plate (Wuxi NEST Biotechnology, Wuxi, China). Briefly, 50 μL/well of DMEM base medium was added from rows C to H in a microtiter plate. Then, 100 μL of ¼-diluted serum was added on the first column and 50 μL was tittered along the rows and the final was discarded, and 50 μL of each virus dilution (10^−1^ to 10^−8^) was distributed from row C to H then incubated for 1 h. Following this, the microtiter plate was seeded with 10^6^/cell/mL BHK-21 cells. Rows A and B were used as a cell control for the virus titration plate and received 100 μL/well of cell suspension and 50 μL/well of DMEM base medium. These rows were incubated at 37 °C and 5% CO_2_ and observed under an inverted microscope (Leica DMil, Wetzlar and Mannheim, Germany) after 72 h (Table 2). Finally, the titer of each virus was determined by the use of the Spearman–Karber formula [24]. Then, the number of positive and negative wells were recorded to determine the 100TCID 50/mL. The 100TCID (tissue culture infective dose) 50/mL refers to the quantity of virus particles per mL that can produce CPE in 50 percent of the inoculated cells.

### 2.5. Solid-Phase Competitive ELISA

Antibodies induced against vaccine structural protein (SP) which were collected from calves at 7, 14, 21, 28, and 56 days post-vaccination were detected by SPCE (kit batch No. D03/May-20) (http://www.izsler.it, accessed on 9 September 2021). Antibodies induced by type A, O, SAT-2, and trivalent vaccines were detected by SPCE according to the manufacturer’s instructions. Appropriately diluted sera (1/10) were incubated for 1 h at room temperature with the known monoclonal antibody trapped antigen coated (specific to the serotype) to the plate. This enabled the specific antibodies present in the sample to bind to the antigen. After that, anti-FMDV serotype-specific mAb, conjugated with peroxidase, was added. This homologous antigen was bound by antibodies of positive sera, whereas in the case of negative sera, the conjugated mAb could bind to the FMDV antigen. After 1 h of incubation at room temperature, the unbound conjugate was removed by washing, and the TMB (3,3′, 5,5′-Tetramethylbenzidine) substrate/chromogenic solution was delivered into wells and incubated for 20 min at room temperature under dark conditions. A colorimetric reaction develops if the conjugated mAb had bound to the virus, i.e., if test serum is negative, while color development is inhibited by positive sera. After the addition of a stop solution, the optical density of the developed color is read by a micro plate photometer. The results were expressed as a PI relative to the maximum optical density at 450 nm. FMDV type A, O, SAT-2 SP antibodies were considered to be absent if the PI was <70% and present in the serum if the PI was ≥70% according to the manufacturer’s protocol. In this case, serum of the calves before vaccination was used as a negative control and the percent of inhibition was undetectable.

### 2.6. One-Dimensional Virus-Neutralization Vaccine-Matching Test

The field isolates were assessed for their serological relationships to the reference vaccine strain of A/ETH/6/2000, O/ETH/38/2005, and SAT-2/ETH/64/2009 against antisera that were raised in calves. It was performed using the one-dimensional VNT, according to the standard protocol of the WOAH [21] manual. Briefly, the log virus dilution having 100TCID_50%_ was chosen and two more virus dilutions corresponding to ±0.5 log_10_ (below and above the 100TCID_50%_) were prepared. For each test and vaccine virus, a chequer-board titration was performed of the virus against vaccine serum along with a back-titration of the virus alone on a Microtest^TM^ 96-well tissue culture polystyrene Nunc plate (Wuxi NEST Biotechnology). 

The one-dimensional virus-neutralization test was performed following the standard protocol of the WOAH Manual of Diagnostic Tests and Vaccines for Terrestrial Animals [21]. Three virus dilutions (100TCID_50%_ and two more virus dilutions ±0.5 log_10_ 100TCID_50%_) were prepared. Antibody titration of the test samples was performed from 1/8 to 1/4096 dilutions (Table 2). In the same fashion, the positive control was also tittered downwards on the respective wells. Then, 50 µL of virus suspension with 100TCID_50%_ of pre-determined log for each vaccine strain virus log_10_ (A), 2.5, (2.5 (O), 1.5 (SAT-2) was added to wells based on their serotype and incubated at 37 °C for 1 h. Finally, 50 µL of cell suspension was added to the well. Then, the cytopathic effect was observed after 72 h and the antibody titer was calculated as per the reciprocal of the highest serum dilution to neutralize 100TCID_50%_ of the virus. Then, the result was expressed as the log_10_ VNT. The virus control and the negative control were supposed to show CPE, whereas in the positive control CPE, this is not expected at the initial, second and third dilution but is at the last dilution. The cell control must contain an intact confluent cell monolayer.

### 2.7. The r1-Value Determination

The r1-value is a way of computing the serum titer of the heterologous strain against the serum titer of the homologous strain (Equation (1)). To compute the r1-value, in total, three antibody titrations were conducted for each sample. This was based on the recommendation of Rweyemamu to increase the confidence and reliability of the test. The titer of the vaccine serum against 100TCID_50%_ of each virus could then be estimated by arithmetic mean. The relationship between the field isolate and the vaccine strain was then expressed as an r1-value as described for the vaccine-matching test by VNT (Equation (1)). In the case of neutralization test, r1-values greater than or equal to 0.3 indicate that the field isolate is sufficiently similar to the vaccine strain. In that use of the vaccine, it is likely to confer protection against challenges with the field isolate [24]. The range of the computed r1-value (‘r1’) = 0.3–1.0 indicates a reasonable degree of cross-protection between the two viruses (field and vaccine), whereas values below 0.3 indicate the need of other strains to develop/include in the vaccine [24].

(1) $$r1-\mathrm{value}=\frac{\mathrm{reciprocal}\;\mathrm{arthmetic}\;\mathrm{mean}\;\mathrm{titer}\;\mathrm{of}\;\mathrm{post}-\mathrm{vaccinal}\;\mathrm{serum}\;\mathrm{against}\;\mathrm{field}\;\mathrm{strain}}{\mathrm{reciprocal}\;\mathrm{arthmetic}\;\mathrm{mean}\;\mathrm{titer}\;\mathrm{of}\;\mathrm{postvaccinal}\;\mathrm{serum}\;\mathrm{against}\;\mathrm{vaccine}\;\mathrm{strain}}$$

## 3. Data Analysis and Management

Data were stored in Excel (version: 14.0.4734.1000), and median and confidence intervals were calculated. Graphs were generated using Excel. The vaccine immune responses quantified by SPCE and VNT were associated with the following: days post-vaccination, serotypes, and dose of the vaccine. The data obtained from one-dimensional virus neutralization assay were also stored in the Microsoft Office Excel spreadsheet. Next, log antibody titers and arithmetic mean of r1-values were calculated. A logistic regression model was used to relate the probability of protection to VNT. Specifically, the probability (p) that an animal with a titer T of VNT was protected after the challenge was given by pred = exp(xb)/(1 + exp(xb)), where a is the intercept and b is the slope. This was used to explore and see the level of protection for a given virus-neutralization titer.

## 4. Results

### 4.1. Antibody Detection against NSP and SP by ELISA

**Figure 1:**
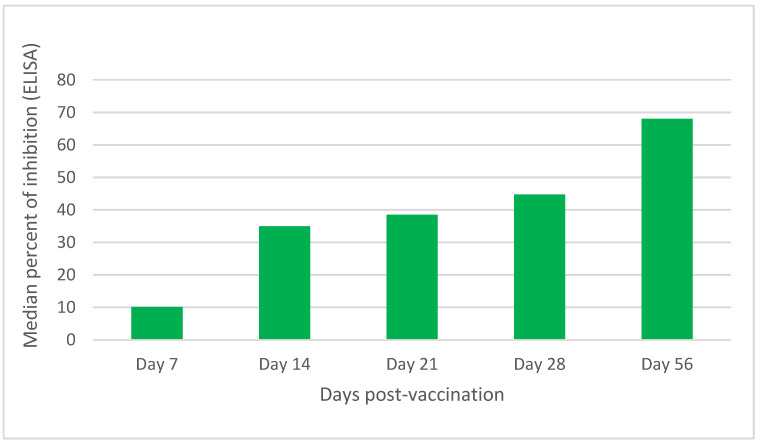
Calves vaccinated with 4 mL trivalent vaccine (serotype O, A, and SAT-2) (N = 5).

**Figure 2:**
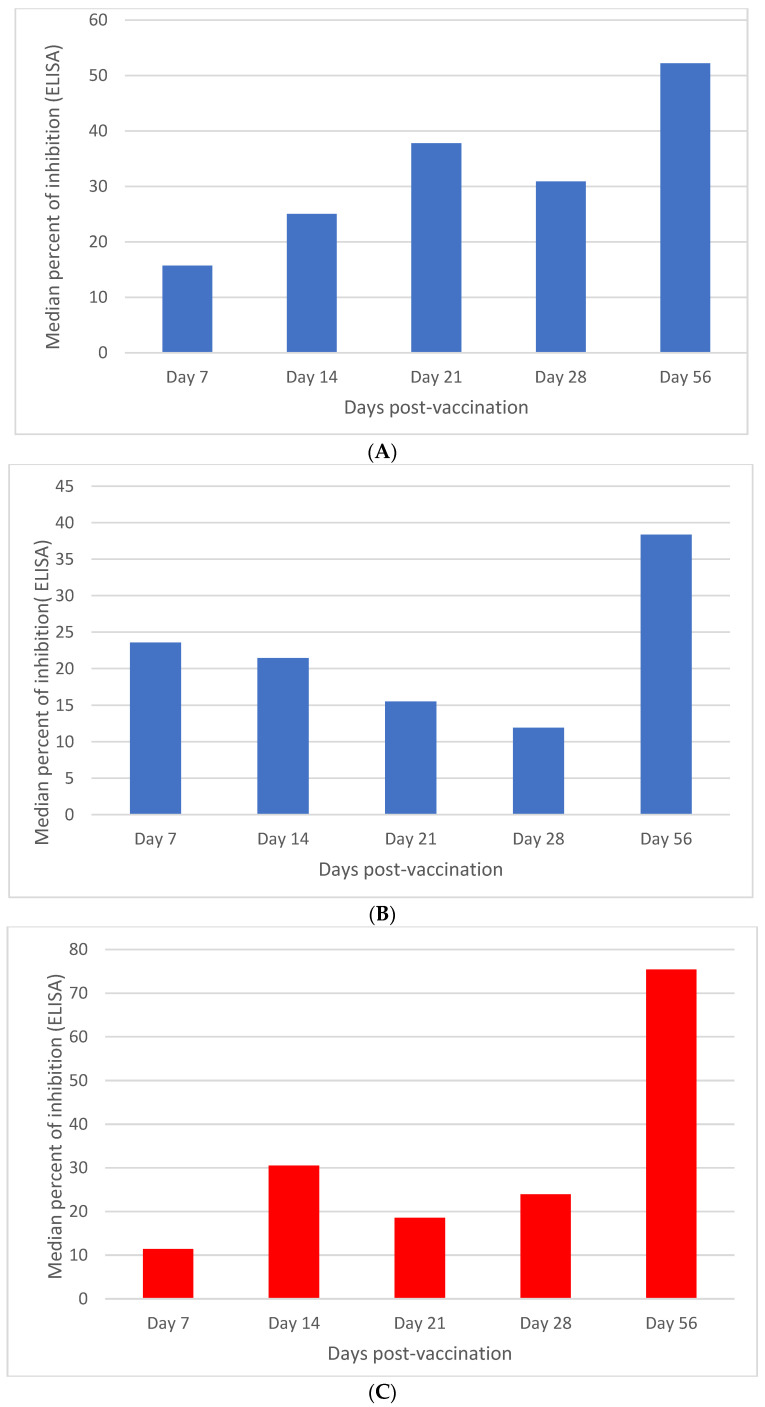

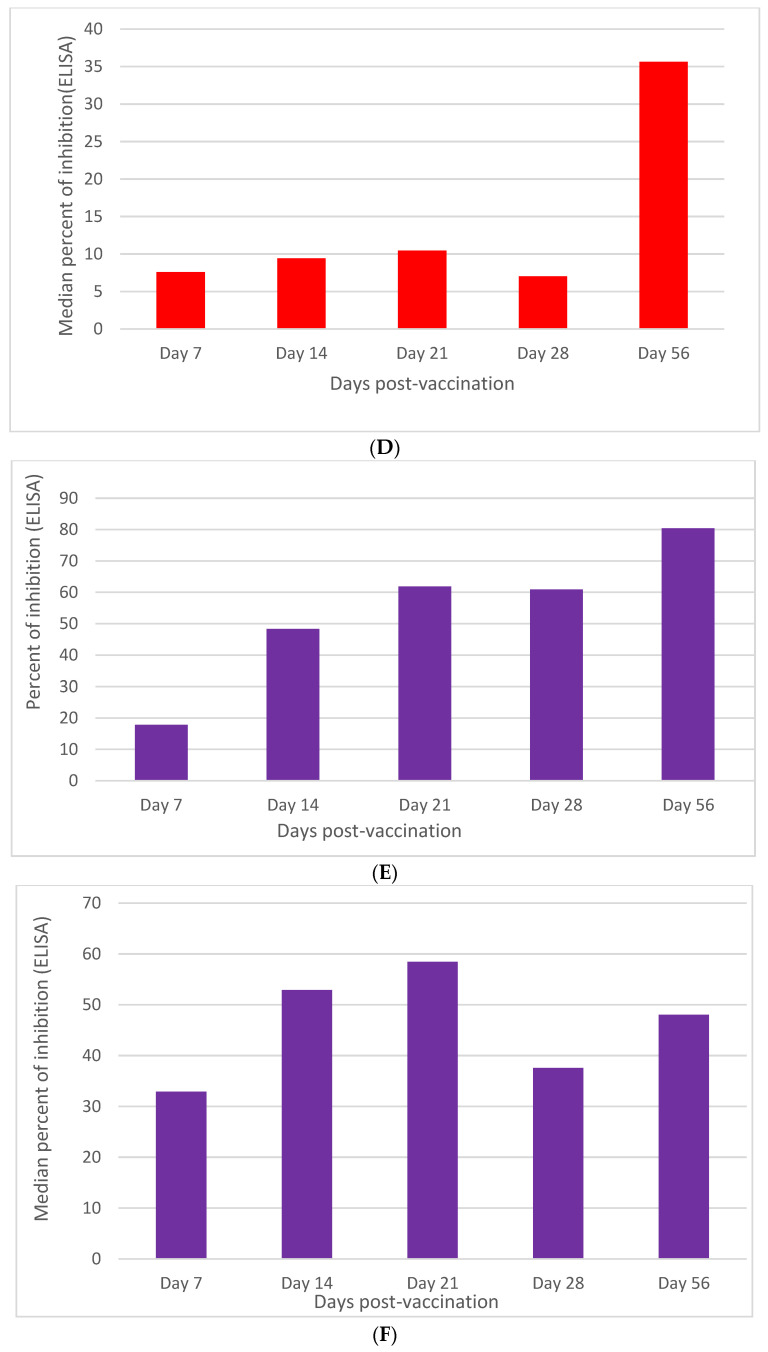
Calves vaccinated with 4 mL and 2 mL doses of vaccine of serotype A, O, and SAT-2 (N = 5). For all, the *Y* axis shows the percent of inhibition of the antibodies against structural proteins of FMDV measured by solid-phase competitive ELISA (SPCE). The *X* axis indicates the days post-vaccination where the serum was analyzed for antibodies against structural proteins. Panel (**A**,**B**) received 4 mL and 2 mL doses of serotype A vaccine. Panel (**C**,**D**) received 4 mL and 2 mL doses of serotype O vaccines. Panel (**E**,**F**) received 4 mL and 2 mL doses of serotype SAT-2 vaccine. Note the increase in the percent of inhibition from days 7–28 (after initial vaccination) to day 56 (28 days after booster) after vaccination.

Serological tests of day zero were conducted to check for antibodies against the non-structural proteins of FMDV. It was found that all animals were sero-negative for antibodies against NSP of FMDV. Post-vaccination sero-monitoring was performed by SPCE to evaluate the presence of antibodies against SP of the vaccine strain. The trend of the immune response was seen to increase from 7 d.p.v. to 56 d.p.v. in calves that received a 4 mL dose of the vaccine irrespective of the serotype (Figure 1 and Figure 2). For the trivalent (containing O, A, and SAT-2) vaccine, 68.5 (CI [48.7, 82.3]) median percent of inhibition was recorded on the 56th day post-vaccination. From the vaccinated calves, 50% showed above 70 percent of inhibition (Figure 1). For calves that received a 4 mL vaccine on the 56th day post-vaccination, the median percent of inhibition for serotype A was 52.20 CI [32.50, 77.13] median percent of inhibition. From the vaccinated calves, only 20% (1/5) showed above 70 percent of inhibition. Similarly, for serotype O, 75.4 CI [55.60, 85.14] median percent of inhibition was recorded. From the vaccinated calves, 60% (3/5) showed above the 70 percent of inhibition. For serotype SAT-2, 80.42 CI [52.48, 105.55] percent of inhibition was recorded. From the vaccinated calves, 80% (4/5) percent of inhibition above 70 was recorded (Figure 2 and Table 3). At day 28 post-vaccination, the percentage of positive calves by SPCE was 20% (1/5), zero percent, and 20% (1/5) of the calves for A, O, and SAT-2 serotype vaccines, respectively. Exceptional to the other calves vaccinated with the vaccine containing O and A serotypes of FMDV, 20% of SAT-2-vaccinated calves at day 7, 14, and 21 days post-vaccination were positive, as determined by SPCE. The increase in antibody titer from days 28 to 56 was statistically significant (*p* < 0.05). On the other hand, for a two-milliliter dose in vaccinated calves, the increase in immune response was seen at 14 d.p.v. (Figure 2). A booster dose at day 28 was given to the calves and on day 56, the serum sample was checked for antibodies against the SP FMDV vaccine. The antibody titer gave 38.33, 35.65, and 48, percent of inhibition for serotype, A, O, and SAT-2 vaccines (Figure 2). This increase was statistically significant at *p* < 0.05 concerning days post-vaccination. But, in this case, there were no calves above the cutoff point of the test of 70% of inhibition.

The *Y* axis shows the percent of inhibition of the antibodies against structural proteins of FMDV measured by solid-phase competitive ELISA (SPCE). The *X* axis indicates the days post-vaccination where the serum was analyzed for antibodies against structural protein. Note the increase in the percent of inhibition from days 7–28 (after initial vaccination) to day 56 (after 28 days of booster) after vaccination.

### 4.2. Virus Neutralization

**Figure 3:**
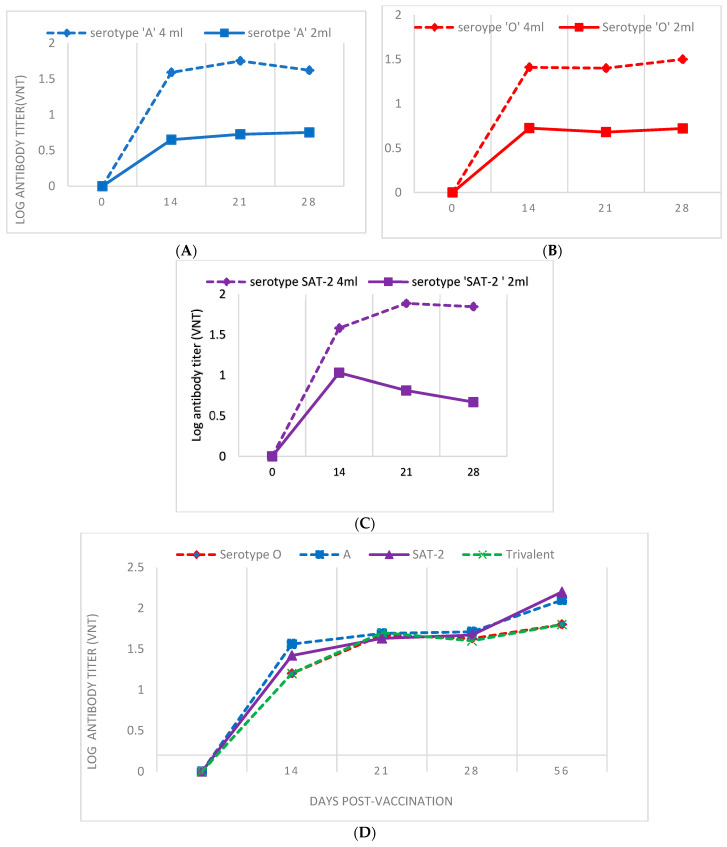
Virus-neutralization test of the post-vaccine serum against the vaccine strain (pooled). For all, the *Y* axis shows the induced antibody measured by virus-neutralization test (VNT). The *X* axis indicates the days post-vaccination where the serum was analyzed. Blue color line: received 4 mL; and red color line: received 2 mL dose. Panel (**A**) for serotype A vaccine. Panel (**B**) for serotype O vaccine. Panel (**C**) for serotype SAT-2 vaccine. Panel (**D**) for trivalent vaccine. Note the increased log antibody titer from days 0 to 28 of vaccination.

Known titers of FMDV for all vaccine strains (A, O, and SAT-2) were used for serum neutralization on BHK-21 cell lines. The cells in the positive-control wells, wherein the virus has been neutralized, were devoid of CPE. In the negative-control and virus-control wells, the virus has not been neutralized; cells show CPE and wells contain damaged cell suspensions. The average log_10_ antibody titer of calves vaccinated with 4 mL FMDV vaccine of serotype A showed a log antibody titer of 1.6, 1.7, and 1.6 at 14, 21, and 28 days post-vaccination against the homologous virus (Figure 3A), respectively. On the other hand, calves that received a 2 mL dose of FMD vaccine containing serotype A gave log antibody titers of 0.65, 0.72, and 0.75 at 14, 21, and 28 days post-vaccination, respectively (Figure 3A). The log antibody titer for serotype O at 4 mL (1.41, 1.4 and 1.5) and 2 mL (0.72, 0.68 and 0.72) doses was recorded (Figure 3B). For the SAT-2 serotype 4 mL dose, the log antibody titer was 1.5, 1.9, and 1.8, whereas for the 2 mL dose, it was 1.03, 0.81, and 0.67, respectively (Figure 3C). Accordingly, for the serotype A trivalent vaccine, the average induced log_10_ virus-neutralizing antibody titer was 1.56, 1.69, 1.71, and 2.1, whereas for serotype O, the 4 mL trivalent vaccine induced, on average, 1.20, 1.67, 1.62, and 1.8 log_10_ virus-neutralizing antibody titers. For serotype SAT-2, the average induced log_10_ virus neutralizing antibody titer was 1.42, 1.63, 1.67, and 2.2 (Figure 3D). All antibody titers were measured 14, 21, and 28 days post-vaccination. But, day zero antibody titers against structural proteins of the virus were undetectable, as expected (Figure 3). The antibody titer was also compared between groups of calves that received a 4 mL dose of FMD vaccine. This shows that there was an increase in all groups of calves that received the vaccine. Calves that received serotype SAT-2 and O booster dose sharply increased their titer. In general, the trend in all vaccinated animals indicates increasing antibody titer following the first dose. Further, the homologous and heterologous virus-neutralization capacity of the post-vaccination antiserum was also evaluated and proved using the one-dimensional virus-neutralization test, though all the tested field strains were not neutralized by the post vaccinal serum of A and O serotype.

### 4.3. Vaccine-Matching Test

The extent of in vitro cross-neutralization of serotype A FMDV field isolates against the vaccine strain was tested using 21-day-post-vaccinal antiserum. Accordingly, the r1-value (arithmetic mean titer) was calculated to check whether the elicited immune response was strong enough to neutralize and matched with field strains of FMDV. For post-vaccinal antiserum raised against A/ETH/06/2000, the r1-values of the field isolates were between 0.25 to 0.40 (average, 0.30 and STDV ± 0.049) (N = 3) (Table 4). In this study, 70% (14/20) of serotype A field isolates had an r1-value greater or equal to 0.30, suggesting a good serological match of the vaccine strain with the majority of the serotype A FMDV field strain tested.

The antigenic relationship of serotype O field isolates with the currently used vaccine strain O/ETH/38/2005 was also determined. Sixty-two percent (23/37) of the field isolates had r1-values greater or equal to 0.3, suggesting a good serological match of the vaccine strain with the majority of the FMDV field strain tested. For post-vaccinal antiserum raised against O/ETH/38/2005, the r1-values of the field isolates were between 0.25 to 0.40 (average, 0.30 and STDV ± 0.048) (N = 3) (Table 4). 

**Figure 4:**
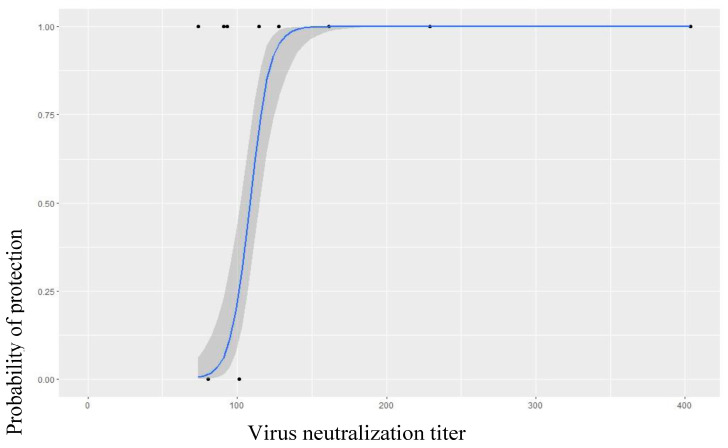
Predicted probability of a post-vaccinal serum neutralizes/protects a field heterologous virus and its dependence on the virus-neutralization antibody titer. The graph shows the probability of protection (*Y* axis) and the virus-neutralization titers (*X* axis). The graph started to rise at 120 titer.

Finally, the antigenic relationship of the four SAT-2 serotype field isolates with the currently used vaccine strain SAT-2/ETH/64/2009 was determined. All of the field isolates had an r1-value greater than 0.3 with the vaccine strain (Table 4). For post-vaccinal serum raised against SAT-2/ETH/64/2009, the serological relationship was between 0.35 and 0.50 (average, 0.40 and STDV ± 0.06) (N = 3). In this serotype also, all SAT-2 field strains tested had an r1-value greater or equal to 0.30, suggesting a good serological match of the vaccine strain with the tested FMDV field strain The predicted probability of protection of a vaccinated animal against field virus challenge was evaluated by an in vitro virus-neutralization test and predicted to be above 120 titer of antibody (Figure 4). In this study, we found 70, 62 and 100 percent serological match of the field strain tested with the post-vaccinal serum used. For SAT-2, it can be concluded that there is a good match and sufficient protection, though further research is required that includes more isolates, whereas for serotype A and O, we recommend further research that encompasses more filed isolates/vaccination strategy/improvement in the vaccine strain used because, according to Mahapatra et al. [29], 80% of the field strains should match with the vaccine strain in use, which is not the case in our condition.

## 5. Discussions

This study tried to assess the immune response and virus-neutralization capacity of the post-vaccinal serum collected from calves vaccinated with vaccines from the National Veterinary Vaccine Institute, Ethiopia. The post-vaccination monitoring protocol described previously was followed to evaluate immune responses induced by vaccination and to estimate the immunity of the population targeted for protection by vaccination and all tests were performed according to WOAH recommendations [30] This experiment utilized 15 calves, six-to-nine-month-old Holstein Friesian calves vaccinated with 2 mL and 15 calves vaccinated with 4 mL doses for serotype A, O, and SAT-2. In addition, a trivalent FMDV vaccine was also given to five calves and four calves were kept as a control. All calves were tested free from antibodies against FMD using NSP competitive ELISA. In our study, the immune response was seen to have a statistically significant increase (*p* < 0.05) from 7 d.p.v. to 56 d.p.v. in calves that received a 4 mL dose of the vaccine irrespective of the serotype. The percent of inhibition obtained here in individual calves was lower than the standard percent of inhibition stated by the test kit, but after receiving booster dose, the median percent of inhibition was found to be higher for O, SAT-2 and trivalent vaccines. For serotype A, the percentage of inhibition was still below 70% (http://www.izsler.it, (accessed on 10 October 2021) FMD structural-protein ELISA).

In our study, antibody titer measured using a virus-neutralization test was 1.7, 1.4 and 1.8 for serotype A, O and SAT-2, respectively. The report of Barnett et al. [31] correlated the immune response measured with protection and documented that log_10_ antibody titers of 1.5, 1.6, and 1.4 for serotype A, O, and SAT-2, respectively, are needed to confer protection for 50 percent of the animals and log_10_ antibody titers of 2.1 were shown to confer protection for 95% of the animals. As such, the data of the present study suggest that a single administration of each monovalent vaccine may protect at least 50% of the animals against FMD for a limited period but a booster vaccination may be necessary to protect for a longer period. This is also supported by predicted probability logistic regression, as 120 antibody titer of day 56 post-vaccination was indicated to protect 50% of the animals vaccinated.

The result of our study was also in agreement with El-Bagoury et al. [32] who found that protective neutralizing serum antibody titer started at the first month post-vaccination with a mean serum-neutralizing antibody titer of 1.7 log_10_ for serotype “A/Egypt/2006”, and 1.6 log_10_ for serotype “O1/3/93” FMD virus, respectively.

Dose-wise comparison of the immune response between 4 mL (standard dose given for trivalent vaccine) and 2 mL doses was compared. The log antibody titer of 4 mL was in the recommended range, whereas that of the 2 mL dose was far below the recommended range and was in agreement with the justification of a dose-based (antigen/immunogen concentration) significant difference of the FMD vaccine immune response [33].

The short humoral immunity duration of the NVI vaccines might be attributed to the adjuvant type used for the formulation. Hence, the combination of aluminum hydroxide gel and oil-adjuvant formulation confers better cellular and humoral immune response [19].

In the one-dimensional virus-neutralization test (1d-VNT), r1-values for serotypes A, O, and SAT-2 indicated that 70%, 62%, and 100% of the tested field viruses were neutralized. This means that 70% of serotype A field strains tested have a good serological/antigenic relationship with the vaccine strain and the remaining have poor serological/antigenic match with the vaccine strain. This indicates that the serological relationship of the tested serotype A FMDV field strains and the vaccine strains are favorable except for a few isolates (30%).

Similarly, 62% of serotype O field strains have a good serological/antigenic relationship with the vaccine strain. Sixty-two percent serological/antigenic match (mean r1-value 0.30 and STDV ± 0.048) between serotype O field isolates and the vaccine strain (O/ETH/38/2005) identified in this study agree with the finding of Tesfaye et al. [8]. They showed that the vaccine virus O/ETH/38/2005 was antigenically matched to 10 of the 16 serotype O viruses (63%) circulating in Ethiopia between 2011 and 2014. The same authors also reported 11 out of 17 (73%) viruses of serotype O and A that circulated in 2018 showed an antigenic match with the vaccine strain [21]. This indicates that the serological relationship of serotype O FMDV field strains and the vaccine strain are favorable except for a few isolates (38%). On the other hand, the mean r1-value for SAT-2/ETH/64/2009 homologous virus with its heterologous field strain was found to be 0.40 ± 0.06, indicating that the total serological relationship of the tested serotype SAT-2 FMDV field strains with the vaccine strain. This is within the recommended range for SAT-2, but amongst the tested serotype A and O field strains, less than 80% as described by [29] and less than 75% as described by [34] showed a lower percentage of serological relationship with the vaccine strain in use.

In addition, we also showed the predicted probability of protection of 50% at 120 virus-neutralization titer. Our finding agreed with various researchers that stated that r1-values greater than 0.3 were indicative of a serological/antigenic match between the tested field isolates and vaccine strain viruses [3,24,34,35].

This is also supported by other researchers [21] that show, for the vaccine strains A/ETH/6/2000 and O/ETH/38/2005, an r1-value between 0.40 and 0.39 for serotypes A and O, respectively. On the contrary, some isolates had a poor serological/antigenic match between the vaccine strain and field strain (r1-value from 0.2 to 0.3) for both serotype A and O but not for SAT-2. These isolates comprise 30% serotype A and 38% of serotype O field isolates (heterologous). This is in line with the finding of Tesfaye et al. [20] that reported 14% of the field viruses (serotype O) responsible for the outbreak in 2014 had a poor serological/antigenic match with the vaccine strains. On the other hand, in vitro test (VNT) results were challenged by previous studies with the evidence that high-potency FMDV vaccines may still protect against field viruses even when the r1-values are low [36,37].

## 6. Conclusions

FMD is an endemic disease in Ethiopia, with no control practices tried so far in Ethiopia. Our study evaluated the humoral immune response and found a significant level of antibody titer at 21 d.p.v. that neutralized the virus. In addition, the antibody titer measured by solid-phase quantitative ELISA at different days post-vaccination indicates that the vaccine is capable of inducing an antibody response. The result showed that 70%, 62% and 100% of selected/tested circulating FMDV serotype A, O, and SAT-2 are a serologic/antigenic match with the vaccine strains used at NVI. The predicted probability of protection of the vaccine was found to be 50% at 120 virus neutralization titer. This study ascertained that the vaccine in use shows a serological relationship with the tested field strain and the importance of booster dose vaccination to maintain the antibody titer. But, as stated by previous researchers, at least an 80% match is expected to be used as a good vaccine strain. Therefore, for serotype A and O, the vaccine strain should be further tested for more field isolates. This work can be further expanded by investigating the cell-mediated immune response or other parameters such as the Th1 cytokines (INF-γ) that might give a better indication of the adaptive immune response. Though our work is not without limitation and can be expanded by further studies, involving in vivo challenges and countrywide isolate coverage, comparison with the natural infection response and the use of different adjuvant types, our finding pointed out that the locally produced vaccines have a good antigenic coverage or protection at 21 days post-vaccination with a 4 mL dose and a booster dose at day 28 post-vaccination. The strategy to obtain more protection against FMD infection in Ethiopia may necessitate further research/change of vaccination strategy/ improvement in the currently used vaccine to cover a wide range of prevailing genotypes/lineages and induction of sound immune response after vaccination. In addition, a regular vaccine-matching test using post-vaccinal serum is necessary for serotypee O and A FMD virus in Ethiopia. This was also supported by the virus-neutralization capacity of pooled serum from day 21 post-vaccination to their heterologous counterparts. In general, regular vaccine-matching tests supported by in vivo challenges, taking into consideration other factors that affect vaccine potency, like the antigen payload, storage condition, and adjuvant used, as well as the vaccination strategy used, are needed to plan a control strategy for FMDV.

## Figures and Tables

**Table 1:** Experimental layout used for humoral immune response study.

Animal Species	Group	Vaccine Strain	Number of Animals	Vaccine Dose	NSP Status
Bovine	1	O	5	4 mL	Negative
Bovine	2	O	5	2 mL	Negative
Bovine	3	A	5	4 mL	Negative
Bovine	4	A	5	2 mL	Negative
Bovine	5	SAT-2	5	4 mL	Negative
Bovine	6	SAT-2	5	2 mL	Negative
Bovine	7	Trivalent	5	4 mL	Negative
Bovine	control	-	4	-	Negative

**Table 2:** Virus neutralization plate layout used for the one-dimensional vaccine-matching test.

	1:8	1:16	1:32	1:64	1:128	1:256	1:512	1:1024	1:2048	1:4096	11	12
A											CC	CC
B											VC	VC
C											NC	NC
D											PC	PC
E												
F												
G												
H												

Rows: A and B cell control, C–H virus titration and Column 1–10 antibody dilution, 11 and 12 controls, CC—cell control, VC—virus control, NC—negative control, PC—positive control.

**Table 3:** Median percent of inhibition at 56 dpv and the 95% confidence interval.

Serotype	Number of Observations	Median Percent of Inhibition	95% Confidence Interval
A—4 mL dose	5	52.20	[32.50, 77.13]
A—2 mL dose	4	38.38	[30.55, 48.66]
O—4 mL dose	5	75.40	[55.60, 85.14]
O—2 mL dose	3	35.64	[32.58, 48.44]
SAT-2—4 mL	5	80.42	[52.48, 105.55]
SAT-2—2 mL	4	48.00	[35.14, 55.49]
Trivalent—4 mL	4	68.50	[48.70, 82.30]

**Table 4:** Representative serotype A, O, and SAT-2 field isolates r1-value against the respective vaccine strains. All VNT tests were repeated three times.

Sample Code	Serotype	Year of Isolate	r1-Value
ETH/0038	Vaccine Strain	2005	1.00
ETH/0044	O	2013	0.32
ETH/003	O	2015	0.25
ETH/006	O	2015	0.32
ETH/0011	O	2016	0.20
ETH/002	O	2016	0.25
ETH/003	O	2016	0.32
ETH/004	O	2016	0.32
ETH/006	O	2016	0.32
ETH/004	O	2017	0.32
ETH/0017	O	2017	0.32
ETH/0024	O	2017	0.25
ETH/002	O	2017	0.32
ETH/0072	O	2018	0.40
ETH/0073	O	2018	0.32
ETH/0060	O	2019	0.25
ETH/0053	O	2019	0.32
ETH/0092	O	2020	0.32
ETH/0095	O	2020	0.20
ETH/006	Vaccine Strain	2000	1.00
ETH/0019	A	2015	0.32
ETH/0028	A	2016	0.25
ETH0044	A	2016	0.32
ETH/008	A	2017	0.40
ETH/0012	A	2017	0.32
ETH/0040	A	2018	0.25
ETH/0085	A	2018	0.32
ETH/0086	A	2018	0.32
ETH/0087	A	2018	0.25
ETH/0060	A	2019	0.25
ETH/0049	A	2019	0.40
ETH/0045	A	2019	0.32
ETH/0064	Vaccine Strain	2009	1.00
ETH/0037	SAT-2	2015	0.51
ETH/0011	SAT-2	2015	0.40
ETH/0056	SAT-2	2015	0.39
ETH/0018	SAT-2	2015	0.32

## Data Availability

The data presented in this study are available on request from the corresponding author.

## References

[1] Belsham G.J. (1993). Distinctive features of foot-and-mouth disease virus, a member of the picornavirus family; aspects of virus protein synthesis, protein processing and structure. Prog. Biophys. Mol. Biol..

[2] Jamal S.M., Belsham G.J. (2013). Foot-and-mouth disease: Past, present and future. Vet. Res..

[3] Lloyd-Jones K., Mahapatra M., Upadhyaya S., Paton D.J., Babu A., Hutchings G., Parida S. (2017). Genetic and antigenic characterization of serotype O FMD viruses from East Africa for the selection of suitable vaccine strain. Vaccine.

[4] Knowles N.J., Samuel A.R. (2003). Molecular epidemiology of foot-and-mouth disease virus. Virus Res..

[5] Paton D.J., Di Nardo A., Knowles N.J., Wadsworth J., Pituco E.M., Cosivi O., Rivera A.M., Kassimi L.B., Brocchi E., de Clercq K. (2021). The history of foot-and-mouth disease virus serotype C: The first known extinct serotype?. Virus Evol..

[6] Ayelet G., Mahapatra M., Gelaye E., Egziabher B.G., Rufeal T., Sahle M., Ferris N.P., Wadsworth J., Hutchings G.H., Knowles N.J. (2009). Genetic characterization of foot-and-mouth disease viruses, Ethiopia, 1981–2007. Emerg. Infect. Dis..

[7] FAO (2018). The Global Foot-and-Mouth Disease Control Strategy. http://www.fao.org/3/I9857EN/i9857en.PDF.

[8] Tesfaye Y., Khan F., Gelaye E. (2020). Molecular characterization of foot-and-mouth disease viruses collected from Northern and Central Ethiopia during the 2018 outbreak. Vet. World.

[9] Gizaw D., Tesfaye Y., Wood B.A., Di Nardo A., Shegu D., Muluneh A., Bilata T., Belayneh R., Fentie A., Asgdome H. (2020). Molecular characterization of foot-and-mouth disease viruses circulating in Ethiopia between 2008 and 2019. Transbound. Emerg. Dis..

[10] Knight-Jones T.J.D., Rushton J. (2013). The economic impacts of foot and mouth disease—What are they, how big are they and where do they occur?. Prev. Vet. Med..

[11] World Organisation for Animal Health Manual 3: Foot and Mouth Disease Vaccination and Post-Vaccination Monitoring. Proceedings of the Sub-Regional Workshop on Developing Risk-Based Strategic Plan for FMD.

[12] Doel T.R., Mahy B.W.J. (2005). Natural and Vaccine Induced Immunity to FMD. Foot-and-Mouth Disease Virus.

[13] Graham J. (2020). BelshamTowards improvements in foot-and-mouth disease vaccine performance. Acta Vet. Scand..

[14] Jamal S.M., Shah S.I., Ali Q., Mehmood A., Afzal M., Afzal M., Dekker A. (2014). Proper quality control of formulated foot-and-mouth disease vaccines in countries with prophylactic vaccination is necessary. Transbound. Emerg. Dis..

[15] Brown F. (2003). The history of research in foot-and-mouth disease. Virus Res..

[16] Choi J.H., You S.H., Ko M.K., Jo H.E., Shin S.H., Jo H., Park J.H. (2020). Improved immune responses and safety of foot-and-mouth disease vaccine containing immunostimulating components in pigs. J. Vet. Sci..

[17] Gamaledin W.M., Elsayed E., Hassanin A.I., Mohamed A.A., Shabana W. (2019). Performance of Aluminum Hydroxide Gel and ISA Oils Adjuvanted Foot and Mouth Disease Vaccines. ARC J. Anim. Vet. Sci..

[18] Ayelet G., Soressa M., Sisay T., Belay A., Gelaye E., Jembere S., Skjerve E., Asmare K. (2013). FMD virus isolates: The candidate strains for polyvalent vaccine development in Ethiopia. Acta Trop..

[19] El-Sayed E.I., Mossad W.G., Hassanin A.I. (2020). Immunomodulating effect of different adjuvants informulation of Foot and Mouth disease vaccine relative to its immunogenicity. J. Appl. Vet. Sci..

[20] Tesfaye Y., Khan F., Yami M., Wadsworth J., Knowles N.J., King D.P., Gelaye E. (2020). A vaccine—Matching assessment of different genetic variants of serotype O foot—And—Mouth disease virus isolated in Ethiopia between 2011 and 2014. Arch. Virol..

[21] Tesfaye Y., Khan F., Gelaye E. (2021). Vaccine matching and antigenic variability of foot-and-mouth disease virus serotypes O and A from 2018 Ethiopian isolates. Int. Microbiol. Off. J. Span. Soc. Microbiol..

[22] WOAH Collection, Submission and Storage of Diagnostic Specimens (Version Adopted in May 2013). https://www.woah.org/en/disease/foot-and-mouth-disease/.

[23] WOAH (2021). Terresterial Manual. Foot and Mouth Disease (Infection with Foot and Mouthdisease). https://www.woah.org/fileadmin/Home/eng/Health_standards/tahm/3.01.08_FMD.pdf.

[24] Rweyemamu M.M. (1984). Antigenic variation in foot-and-mouth disease: Studies based on the virus neutralization reaction. J. Biol. Stand..

[25] Das B., Sanyal A., Subramaniam S. (2012). Field outbreak strains of serotype O foot-and-mouth disease virus from India with a deletion in the immunodominant β G-β H loop of the VP1 protein. Arch. Virol..

[26] Subramaniam S., Mohapatra J.K., Das B., Sanyal A., Pattnaik B. (2015). Infection, Genetics and Evolution Genetic and antigenic analysis of foot-and-mouth disease virus serotype O responsible for outbreaks in India during 2013. Infect. Genet. Evol..

[27] Paton D.J., Valarcher J.F., Bergmann I., Matlho O.G., Zakharov V.M., Palma E.L., Thomson G.R. (2005). Selection of foot and mouth disease vaccine strains: A review. Rev. Sci. Tech..

[28] Rweyemamu M.M., Hingley P.J. (1984). Foot and mouth disease virus strain differentiation: Analysis of the serological data. J. Biol. Stand..

[29] Mahapatra M., Upadhyaya S., Aviso S., Babu A., Hutchings G., Parida S. (2017). Selection of vaccine strains for serotype O foot-and-mouth disease viruses (2007–2012) circulating in Southeast Asia, East Asia and Far East. Vaccine.

[30] Ferrari G., Paton D., Duffy S., Bartels C., Knight-Jones T. (2016). Foot and Mouth Disease Vaccination Monitoring Guidelines. http://www.fao.org/3/i5975e/I5975E.pdf.

[31] Barnett P.V., Statham R.J., Vosloo W., Haydon D.T. (2003). Foot-and-mouth disease vaccine potency testing: Determination and statistical validation of a model using a serological approach. Vaccine.

[32] El-Bagoury G.F., El-Habbaa A.S., Heba A.M.B., Halima M.E.-W. (2013). Assessment of immune response to a local inactivated bivalent oil FMD vaccine in calves under field condition. Banha Vet. Med. J..

[33] Sims L.D., Dyrting K.C., Wong K.W. (2000). Serological Response of Pigs to a Standard and Increased Dose of Foot-and-Mouthdisease Vaccine.

[34] WOAH Foot-and-Mouth-, Disease-Chapter 2.1.5. https://www.woah.org/fileadmin/Home/eng/Animal_Health_in_the_World/docs/pdf/2.01.05_FMD.pdf.

[35] Paton D.J., Sumption K.J., Charleston B. (2009). Options for control of foot-and-mouth disease: Knowledge, capability and policy. Philos. Trans. R. Soc. B Biol. Sci..

[36] Nagendrakumar S.B., Srinivasan V.A., Madhanmohan M., Yuvaraj S., Parida S., Di Nardo A., Horsington J., Paton D.J. (2011). Evaluation of cross-protection between O 1 Manisa and O 1 Campos in cattle vaccinated with foot-and-mouth disease virus vaccine incorporating different payloads of inactivated O 1 Manisa antigen. Vaccine.

[37] Brehm K.E., Kumar N., Thulke H., Haas B. (2008). High potency vaccines induce protection against heterologous challenge with foot-and-mouth disease virus. Vaccine.

